# Supplementary figures and images for: N-myc downstream regulated gene 1 (ndrg1) functions as a molecular switch for cellular adaptation to hypoxia
Source: eLife. 2022 Oct 10;11:e74031. doi: 10.7554/eLife.74031 (PMC9550225; doi:10.7554/eLife.74031)

## Slide 1
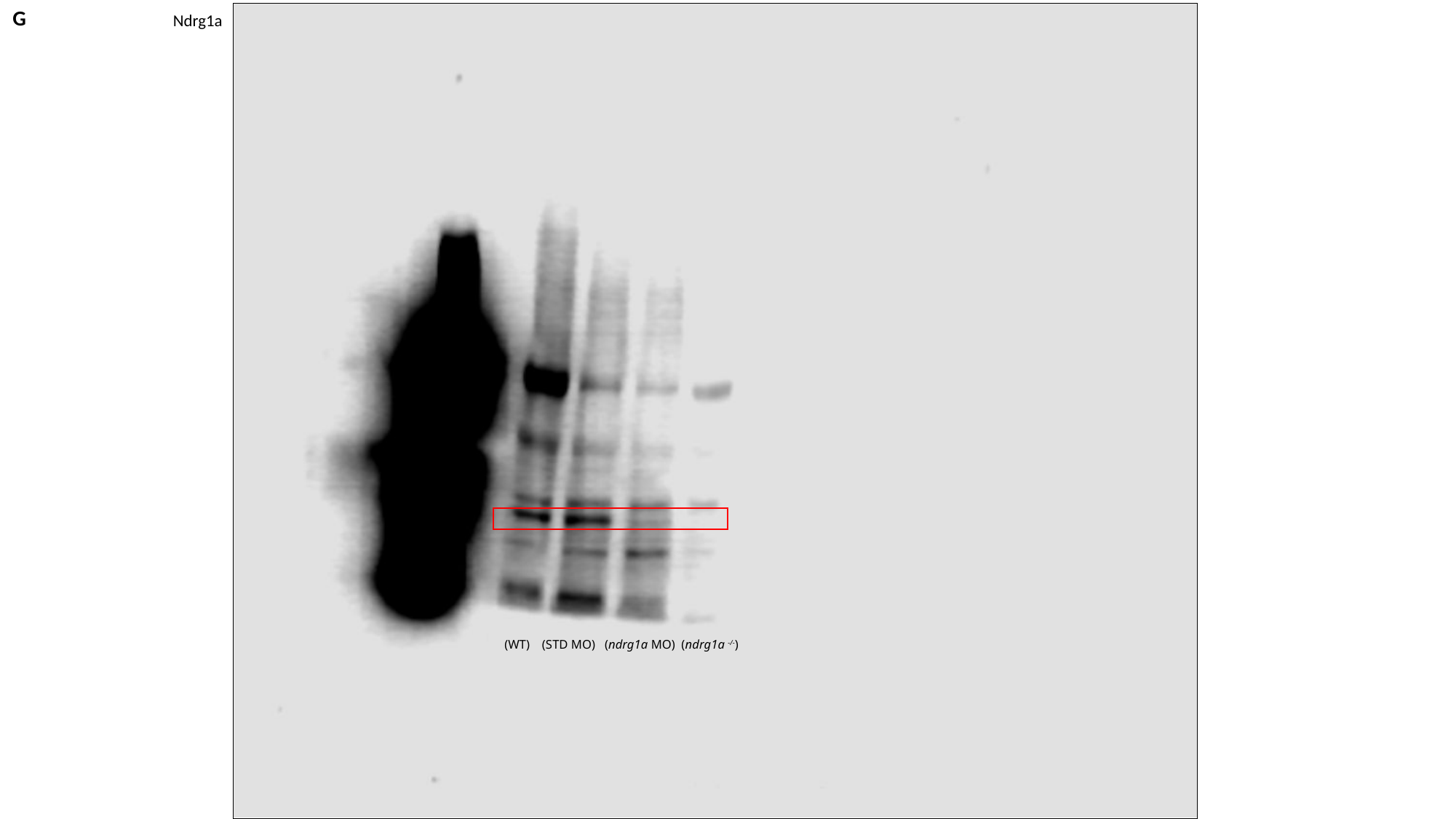

G
Ndrg1a
(WT) (STD MO) (ndrg1a MO) (ndrg1a -/-)

## Slide 2
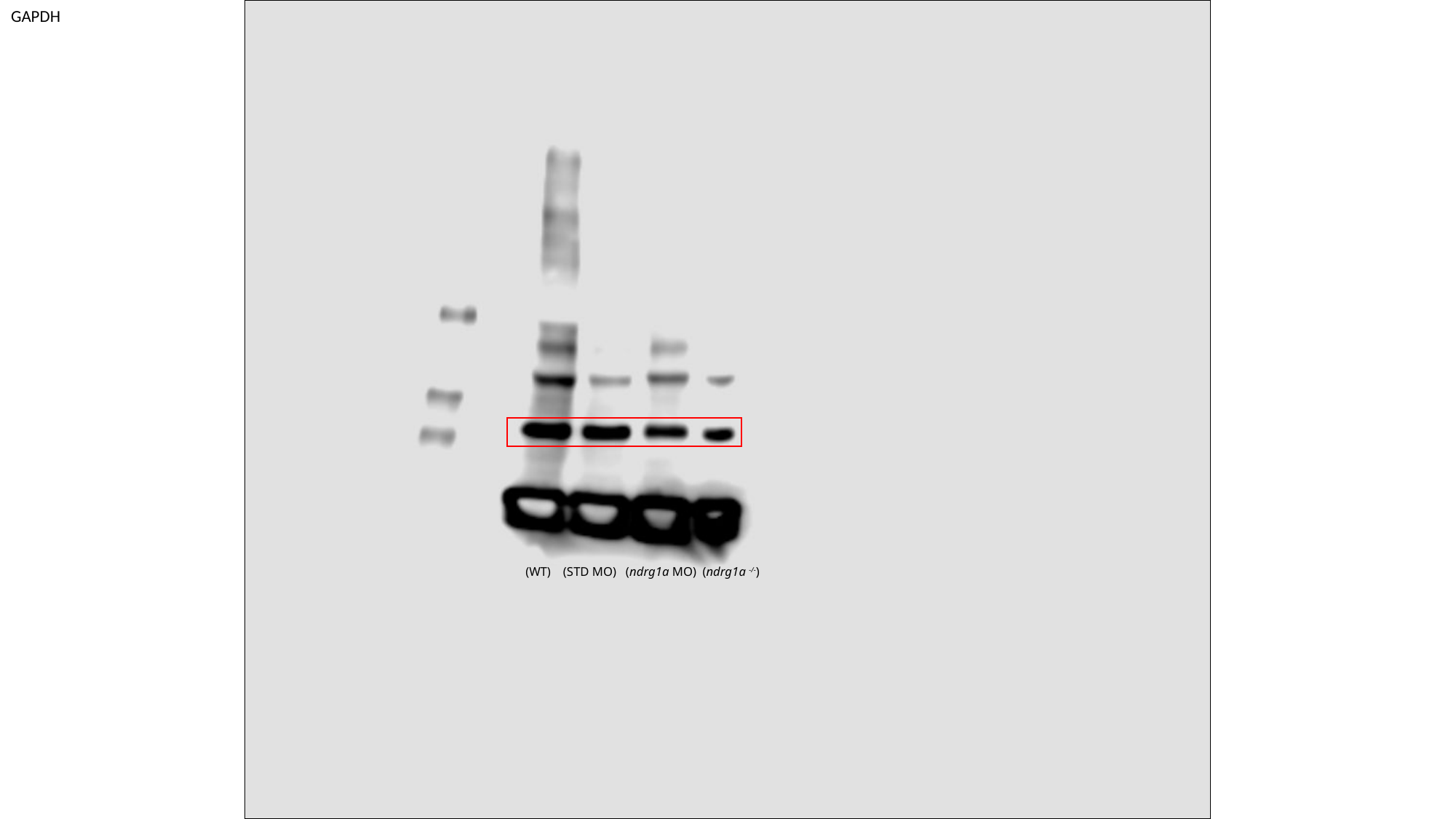

GAPDH
(WT) (STD MO) (ndrg1a MO) (ndrg1a -/-)

Supplement: Figure 1—figure supplement 1—source data 1. [file elife-74031-fig1-figsupp1-data1.zip › Figure 1-figure supplement 1-Source Data 1.pptx]

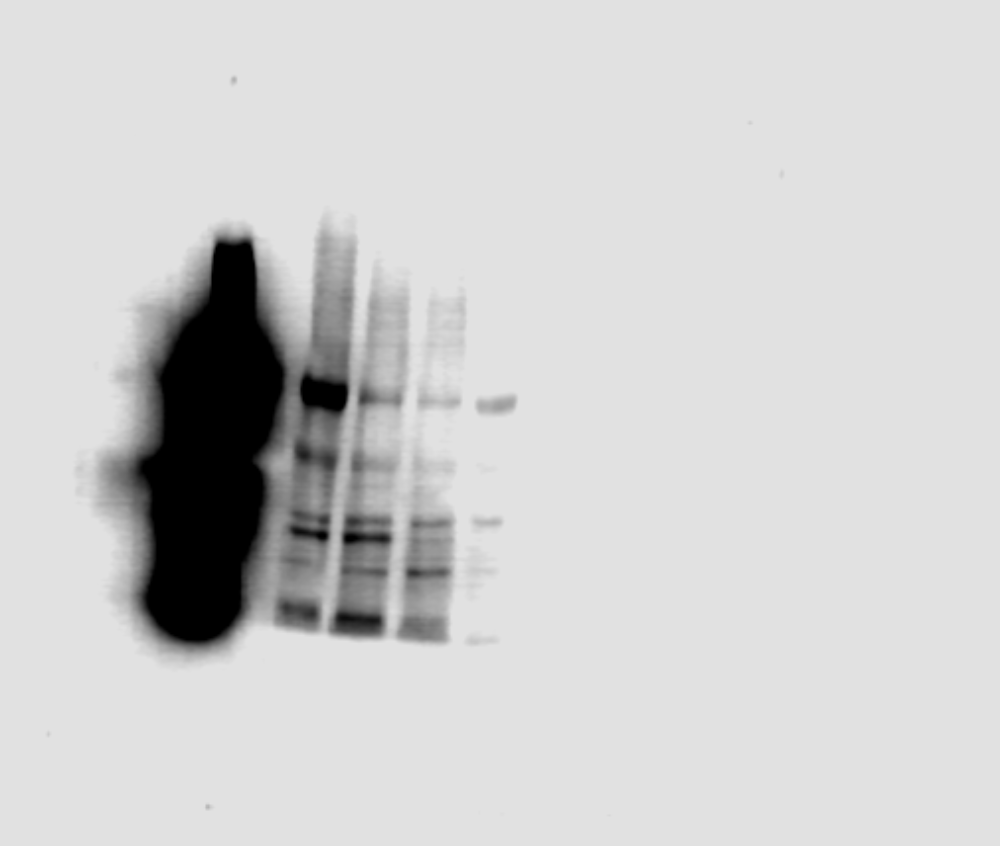

Supplement: Figure 1—figure supplement 1—source data 1. [file elife-74031-fig1-figsupp1-data1.zip › Figure 1-figure supplement 1-Source Data 1-1.tif]

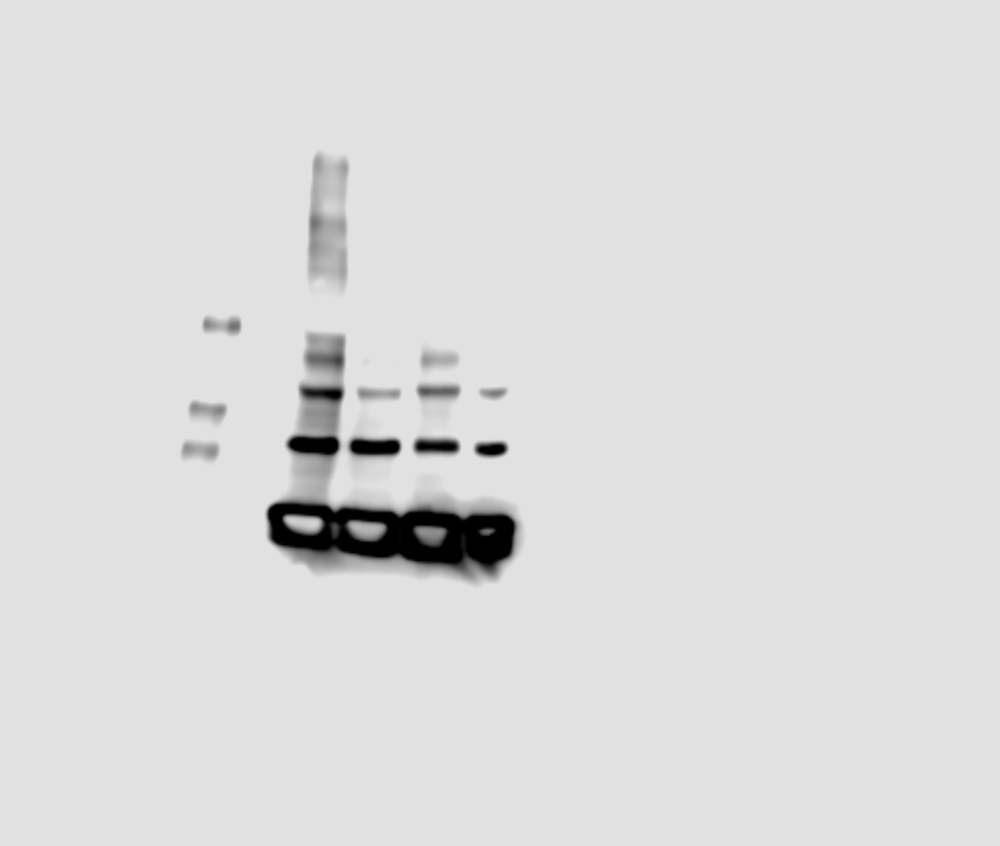

Supplement: Figure 1—figure supplement 1—source data 1. [file elife-74031-fig1-figsupp1-data1.zip › Figure 1-figure supplement 1-Source Data 1-2.tif]
